# Supplementary material for: The Responses to Long-Term Water Addition of Soil Bacterial, Archaeal, and Fungal Communities in a Desert Ecosystem
Source: Microorganisms. 2021 Apr 30;9(5):981. doi: 10.3390/microorganisms9050981 (PMC8147197; doi:10.3390/microorganisms9050981)
Supplement: Supplementary file 1 [file microorganisms-09-00981-s001.zip › microorganisms-1192912-supplementary.pdf]

Table S1. PerMANOVA (pairwise comparisons between treatments) to evaluate variations in the bacterial, archaeal and fungal community structure. Significant p values are in bold.

Group	Bateria		Archaea		Fungi	
	R-Square	p -Value	R-Square	p -Value	R-Square	p -Value
C vs W25	0.400	**0.001**	0.322	0.078	0.142	0.532
C vs W50	0.329	**0.030**	0.478	**0.001**	0.225	0.082
C vs W100	0.456	**0.031**	0.469	**0.030**	0.269	0.057
W25 vs W50	0.208	0.065	0.406	**0.001**	0.135	0.491
W25 vs W100	0.227	**0.001**	0.411	**0.024**	0.134	0.461
W50 vs W100	0.280	**0.024**	0.573	**0.033**	0.287	0.051
C vs W25 vs W50 vs W100	0.422	**0.001**	0.558	**0.001**	0.276	0.054

C = ambient precipitation; W25 = ambient precipitation +25% of local annual mean precipitation; W50 = ambient precipitation + 50% of local annual mean precipitation; W100 = ambient precipitation +100% of local annual mean precipitation.

Table S2. Results of one-way ANOVA of the effects of water addition (W) on the relative abundances of soil bacterial phyla (≥0.1%). The mean value and standard error (n = 4) of each taxon at each water addition-treatment level (C, W25, W50 and W100) are shown in the right columns of the table. p values reflecting statistical significance are shown in boldface. Lowercase letters in the right columns of the table indicate significant differences observed following water addition (p < 0.05).

Domain	Change	Taxa groups	C (%)	W25 (%)	W50 (%)	W100 (%)
Bacteria	Increased	*p_Proteobacteria*	29.7±2.5**bc**	35.9±1.9**ab**	28.0±2.7**c**	39.5±2.0**a**
		p_Acidobacteria	2.0±0.1**c**	2.9±0.3**bc**	3.4±0.6**b**	5.1±0.4**a**
		p_Planctomycetes	0.5±0.0**b**	0.7±0.2**b**	0.9±0.2**ab**	1.3±0.1**a**
		p_Gemmatimonadetes	3.3±0.3**b**	3.5±0.3**ab**	4.4±0.4**a**	3.8±0.2**ab**
		p_ Nitrospirae	0.1±0.0**c**	0.18±0.01**bc**	0.28±0.05**a**	0.26±0.01**ab**
		p_ Deinococcus-Thermus	0.2±0.01**b**	0.6±0.2**a**	0.5±0.1**ab**	0.40±0.0**ab**
	Decreased	*p_Actinobacteria*	43.9±2.1**a**	41.5±1.3**a**	46.7±1.7**a**	34.7±1.9**b**
		p_Firmicutes	8.5±1.5**a**	5.6±0.4**b**	5.3±0.6**b**	4.2±0.3**b**
		p_Bacteroidetes	3.2±0.3**a**	2.5±0.2**ab**	2.3±0.4**b**	2.1±0.1**b**
		p_Thermomicrobia	1.2±0.1**a**	0.8±0.1**b**	0.8±0.0**b**	0.9±0.1**ab**
	Unchanged	*p_Chloroflexi*	4.3±0.4**a**	3.2±0.2**a**	4.1±0.5**a**	4.4±0.4**a**
		p_Cyanobacteria	0.3±0.1**a**	0.2±0.0**a**	0.7±0.3**a**	0.2±0.0**a**
		p_Verrucomicrobia	0.5±0.0**a**	0.5±0.1**a**	0.6±0.1**a**	0.7±0.1**a**
Archaea	Increased	*p_Thaumarchaeota*	81±1.7**b**	84.6±2.2**b**	84.6±2.8**b**	93.3±1.2**a**
		g__Candidatus_Nitrososphaera	15.3±1.7**b**	19.4±0.5**b**	18.7±0.8**b**	21.9±1.6**a**
	Decreased	*p_Euryarchaeota*	18.9±1.7**a**	15.1±2.19**b**	15.2±2.7**b**	6.3±1.0**b**
		o_Thermoplasmatales	18.8±1.7**a**	15.1±2.19**b**	15.2±2.7**b**	6.3±1.0**b**

Table S3 Correlations between dominant phyla of microbial communities (based on Bray-Curtis distances) and soil and/or plant variables.

	**TN**		**TP**		**TC**		**pH**		**NO_3-N**		**Plant coverage**		**Plant species**		**Moisture**		**T**	
	**r**	***P***	**r**	***P***	**r**	***P***	**r**	***P***	**r**	***P***	**r**	***P***	**r**	***P***	**r**	***P***	**r**	***P***
**Bacteria**	0.028	0.427	-0.087	0.729	-0.001	0.491	-0.020	0.574	0.013	0.455	0.015	0.440	-0.014	0.520	0.262	**0.030**	0.116	0.117
Actinobacteria	-0.071	0.679	-0.068	0.702	-0.104	0.766	-0.041	0.583	0.025	0.413	0.045	0.331	-0.011	0.511	0.082	0.257	0.173	*0.054*
Proteobacteria	-0.02	0.542	-0.089	0.757	-0.02	0.526	0.004	0.497	0.018	0.430	0.009	0.426	-0.015	0.511	0.254	**0.034**	0.114	0.157
Firmicutes	0.127	0.221	-0.103	0.769	-0.059	0.553	-0.002	0.456	-0.072	0.621	-0.005	0.462	-0.015	0.528	0.100	0.220	-0.068	0.669
Chloroflexi	0.101	0.217	-0.12	0.839	0.167	0.104	-0.031	0.579	0.021	0.412	-0.122	0.864	0.123	0.107	0.257	**0.026**	-0.012	0.512
Gemmatimonadetes	0.074	0.310	-0.02	0.544	-0.039	0.594	0.049	0.345	0.02	0.388	-0.069	0.717	-0.105	0.872	0.225	**0.044**	0.198	**0.041**
Bacteroidetes	0.023	0.407	0.043	0.338	-0.043	0.576	-0.055	0.628	-0.058	0.649	-0.065	0.694	-0.022	0.543	0.094	0.182	0.11	0.118
Acidobacteria	0.292	**0.023**	0.035	0.344	0.373	**0.013**	-0.049	0.610	0.109	0.182	-0.001	0.468	-0.055	0.709	0.551	**0.001**	-0.021	0.552
Thermomicrobia	-0.265	0.987	-0.139	0.840	-0.143	0.828	0.145	0.187	0.097	0.261	0.032	0.358	-0.112	0.869	-0.115	0.799	0.09	0.188
Planctomycetes	-0.023	0.530	-0.02	0.563	-0.044	0.585	-0.156	0.863	-0.094	0.777	-0.043	0.608	0.105	0.150	0.089	0.202	0.084	0.199
Cyanobacteria	-0.086	0.614	0.005	0.458	-0.181	0.823	0.266	0.112	-0.186	0.879	0.086	0.273	0.045	0.308	-0.174	0.878	0.155	0.107
**Archaea**	0.070	0.287	0.127	0.173	0.141	0.173	0.153	0.157	0.212	*0.082*	0.104	0.205	-0.162	0.955	0.176	0.104	0.282	**0.017**
Thaumarchaeota	-0.077	0.625	0.087	0.272	-0.011	0.461	0.241	*0.088*	0.230	0.102	0.015	0.401	-0.146	0.938	-0.024	0.479	0.309	**0.010**
Euryarchaeota	0.606	**0.001**	0.169	*0.068*	0.669	**0.003**	-0.182	0.945	0.086	0.204	0.230	**0.019**	-0.055	0.706	0.732	**0.001**	-0.003	0.377
**Fungi**	0.072	0.322	0.030	0.399	0.154	0.165	0.219	0.105	0.040	0.344	-0.158	0.916	-0.071	0.720	0.270	*0.054*	0.021	0.383
Ascomycota	0.076	0.306	0.037	0.373	0.159	0.145	0.232	*0.092*	0.069	0.293	-0.231	0.992	-0.081	0.764	0.246	*0.051*	0.010	0.410

p values reflecting statistical significance are shown in boldface. p values reflecting marginal significance are shown in Italic.

Abbrevitions: TC, total carbon; TN, total nitrogen; TP, total phosphorus; T, soil temperature at the depth of 20 cm; Moisture, soil moisture at the depth of 20 cm.

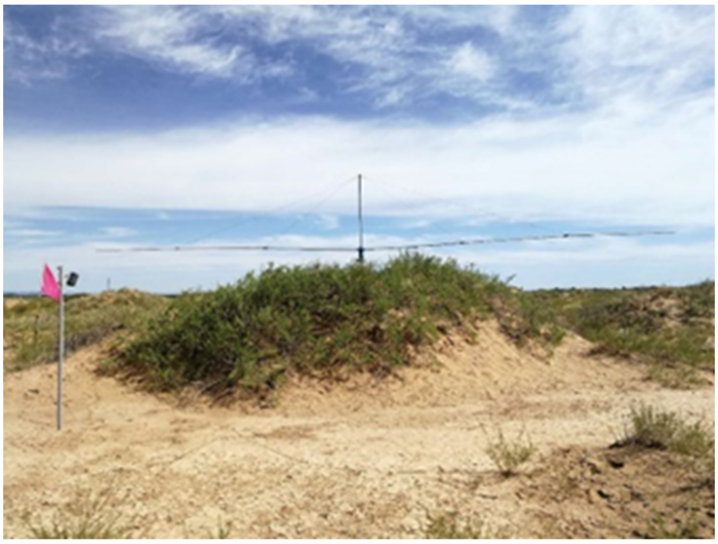

Figure S1 Water addition treatments were conducted using an irrigation system

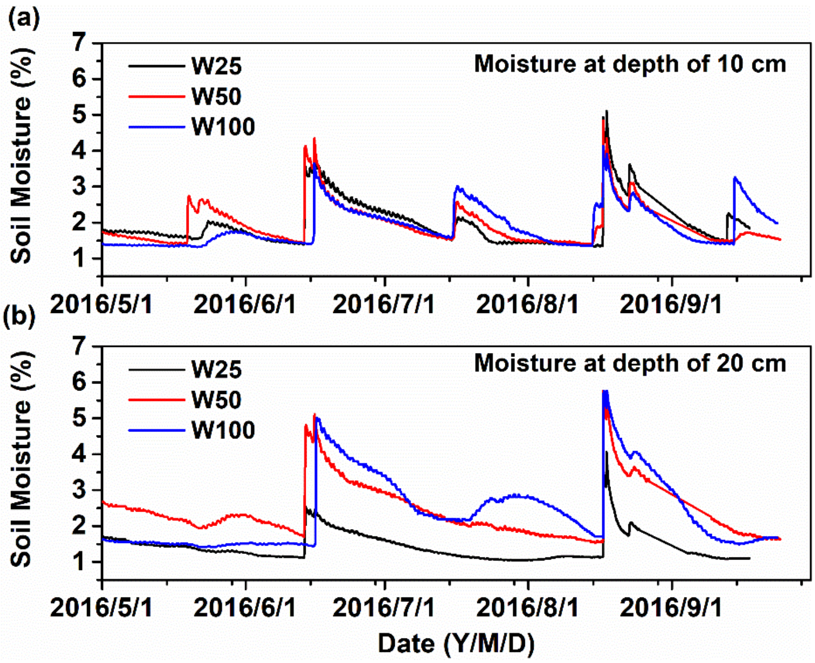

Figure S2 Soil moisture at depth of 10 cm and 20 cm of nabkhas under different water addition treatments. The treatments were equally applied on the 15th of each month from May to September, totally five times, which can be seen from Figure S2a. Soil samples were collected from the topsoil (0-20 cm in depth). Thus, soil moisture which was used in our study was at the depth of 20 cm. From Figure S2b, soil moisture had stabilized since September 20th, 2016. Our soil samples were collected on September 22nd, 2016.

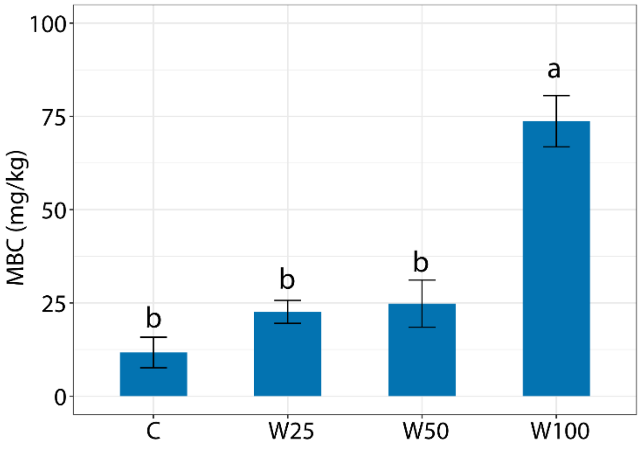

Figure S3 Effects of water addition on the soil microbial biomass carbon (MBC).Means ± SE are presented (n = 4). We used the One-way ANOVA method to detect significant changes between treatments. C = ambient precipitation; W25 = ambient precipitation +25% of local annual mean precipitation; W50 = ambient precipitation + 50% of local annual mean precipitation; W100 = ambient precipitation +100% of local annual mean precipitation.

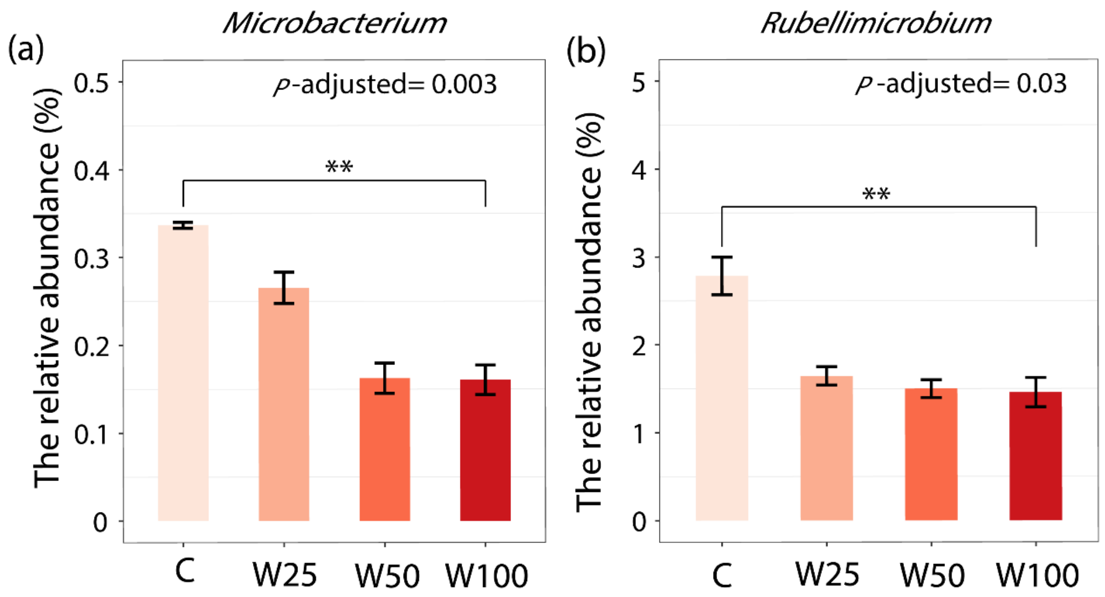

Figure S4 Effects of water addition on the genus of *Microbacterium* (a) and *Rubellimicrobium* (b). Means ± SE are presented (n = 4). We used the One-way ANOVA method to detect significant changes between treatments.**, p < 0.01. C = ambient precipitation; W25 = ambient precipitation +25% of local annual mean precipitation; W50 = ambient precipitation + 50% of local annual mean precipitation; W100 = ambient precipitation +100% of local annual mean precipitation.

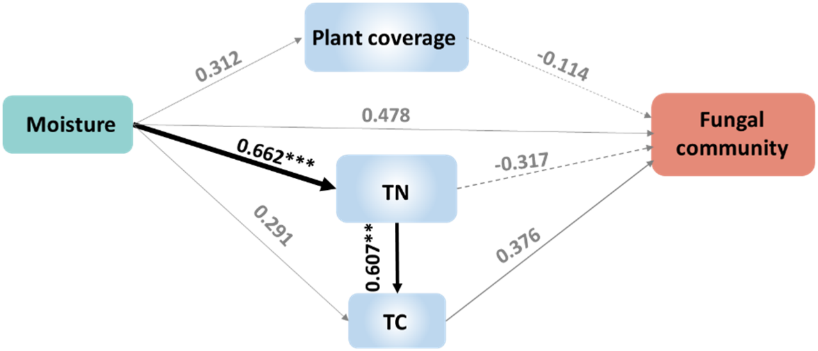

Figure S5 Structural equation modeling showing the relationships between plant/soil properties and the fungal community compositions. Solid arrows indicate positive effects, and the dashed arrow indicates a negative correlation. The standardized path coefficients are adjacent to the arrows and indicate the effect size of the relationship. Arrow widths are proportional to the strength of each relationship. Percentages beside the response variables refer to the proportion of variance explained by the model (R^2). Results of model fitting: χ^2 =0.849, df = 2, p = 0.958; CFI =1.000; AIC =34.085; RMSEA = 0.000, p = 0.960. TN, soil total nitrogen; TC, soil total carbon; Moisture, soil moisture at the depth of 20 cm. $**$, p < 0.01, and $***$, p < 0.001.
